# The Dysregulation of the Monocyte–Dendritic Cell Interplay Is Associated with In-Hospital Mortality in COVID-19 Pneumonia

**DOI:** 10.3390/jcm13092481

**Published:** 2024-04-24

**Authors:** Domenico Galati, Domenico Mallardo, Carmine Nicastro, Serena Zanotta, Ludovica Capitelli, Carmen Lombardi, Bianca Baino, Ernesta Cavalcanti, Silvia Sale, Francesco Labonia, Rita Boenzi, Luigi Atripaldi, Paolo Antonio Ascierto, Marialuisa Bocchino

**Affiliations:** 1 Hematology-Oncology and Stem Cell Transplantation Unit, Department of Hematology and Innovative Diagnostics, Istituto Nazionale Tumori–IRCCS-Fondazione G. Pascale, 80131 Naples, Italy; d.galati@istitutotumori.na.it (D.G.); s.zanotta@istitutotumori.na.it (S.Z.); 2 Unit of Melanoma and Innovative Therapy, Department of Melanoma, Cancer Immunotherapy and Development Therapeutics, Istituto Nazionale Tumori–IRCCS-Fondazione G. Pascale, 80131 Naples, Italy; d.mallardo@istitutotumori.na.it (D.M.); p.ascierto@istitutotumori.na.it (P.A.A.); 3 Clinical Biochemistry Unit, AORN dei Colli, Ospedale Monaldi, 80131 Naples, Italy; carmine.nicastro@ospedalideicolli.it (C.N.); silviasale@libero.it (S.S.); rita.boenzi@ospedalideicolli.it (R.B.); luigi.atripaldi@ospedalideicolli.it (L.A.); 4 Respiratory Medicine Division, Department of Clinical Medicine and Surgery, Federico II University, Monaldi Hospital, 80131 Naples, Italy; ludovica.capitelli@gmail.com (L.C.); carmen.lombardi@icsmaugeri.it (C.L.); b.baino@studenti.unina.it (B.B.); 5 Laboratory Medicine Unit, Istituto Nazionale Tumori–IRCCS-Fondazione G. Pascale, 80131 Naples, Italy; e.cavalcanti@istitutotumori.na.it (E.C.);

**Keywords:** COVID-19, SARS-CoV-2, dendritic cells, monocytes CD169, gene profile analysis

## Abstract

**Background:** The monocyte–phagocyte system (MPS), including monocytes/macrophages and dendritic cells (DCs), plays a key role in anti-viral immunity. We aimed to analyze the prognostic value of the MPS components on in-hospital mortality in a cohort of 58 patients (M/F; mean age ± SD years) with COVID-19 pneumonia and 22 age- and sex-matched healthy controls. **Methods:** We measured frequencies and absolute numbers of peripheral blood CD169^+^ monocytes, conventional CD1c^+^ and CD141^+^ (namely cDC2 and cDC1), and plasmacytoid CD303^+^ DCs by means of multi-parametric flow cytometry. A gene profile analysis of 770 immune-inflammatory-related human genes and 20 SARS-CoV-2 genes was also performed. **Results:** Median frequencies and absolute counts of CD169-expressing monocytes were significantly higher in COVID-19 patients than in controls (*p* 0.04 and *p* 0.01, respectively). Conversely, percentages and absolute numbers of all DC subsets were markedly depleted in patients (*p* < 0.0001). COVID-19 cases with absolute counts of CD169^+^ monocytes above the median value of 114.68/μL had significantly higher in-hospital mortality (HR 4.96; 95% CI: 1.42–17.27; *p* = 0.02). Interleukin (IL)-6 concentrations were significantly increased in COVID-19 patients (*p* < 0.0001 vs. controls), and negatively correlated with the absolute counts of circulating CD1c^+^ cDC2 (r = −0.29, *p* = 0.034) and CD303^+^ pDC (r = −0.29, *p* = 0.036) subsets. Viral genes were upregulated in patients with worse outcomes along with inflammatory mediators such as interleukin (IL)-1 beta, tumor necrosis-α (TNF-α) and the anticoagulant protein (PROS1). Conversely, surviving patients had upregulated genes related to inflammatory and anti-viral-related pathways along with the T cell membrane molecule CD4. **Conclusions:** Our results suggest that the dysregulated interplay between the different components of the MPS along with the imbalance between viral gene expression and host anti-viral immunity negatively impacts COVID-19 outcomes. Although the clinical scenario of COVID-19 has changed over time, a deepening of its pathogenesis remains a priority in clinical and experimental research.

## 1. Introduction

At the end of 2019, many cases of human infection in Wuhan, China, were associated with a novel coronavirus termed severe acute respiratory syndrome Ccoronavirus (SARS-CoV-2). The pandemic that followed immediately challenged the whole medical and scientific community because of the extreme seriousness of the clinical picture associated with it. Initially, the clinical presentation of Coronavirus disease (COVID-19) ranged from asymptomatic to mild upper-respiratory illness, severe bilateral pneumonia, acute respiratory distress syndrome (ARDS), disseminated thrombosis, multi-organ failure and death [1,2,3,4]. There was no doubt that the lung was the preferred target of the virus, especially in the first waves of the pandemic. The clinical presentation of the disease has changed over time with the emergence of genetic variants and following vaccination campaigns. Clinical and scientific evidence overall suggests that SARS-CoV-2 infection is a systemic disorder, the outcome of which is strongly conditioned by the state of the immune system of the host. 

To date, much useful information has been obtained on the mechanisms of infection since the first observations of the virus entering the target cells through the expression of a characteristic spike protein (S protein) binding to the ACE-2 receptor [5,6,7]. The resulting exuberant activation of the inflammatory response, the so-called cytokine storm, has represented the most critical piece of the pathogenesis puzzle that still remains to be fully understood. Monocytes and macrophages are natural immune cells that, together with dendritic cells (DCs), which are professional antigen-presenting cells (APC), constitute the monocyte–phagocyte system (MPS). There is much evidence that the MPS plays a key role in protecting against virus infections, including SARS-CoV-2 [2,8,9,10,11] 

Monocytes and monocyte-derived macrophages were reported to be significantly increased in the peripheral blood and broncho-alveolar lavage (BAL), respectively, of patients hospitalized with COVID-19 pneumonia [12,13,14]. In particular, monocytes and macrophages expressing type I interferon-inducible receptor CD169 have been associated with severe COVID-19 [15,16,17,18]. The engagement of these cells upon SARS-CoV-2 infection was found to actively contribute to the expression of pro-inflammatory cytokines and tissue damage [9,12,19,20,21,22,23,24]. DCs are essential in initiating and regulating adaptive immune responses. Several studies have investigated the interaction between SARS-CoV-2 and DCs, showing that SARS-CoV-2 can directly infect them and/or impair their number and function [25,26,27,28,29]. As for monocytes and macrophages, any dysregulation of the DC compartment resulted in an uncontrolled inflammatory reaction along with tissue damage, all suggestive of a host’s failure to defend itself [25]. These findings are especially important when considering that the interaction between DCs and CD169^+^ monocytes plays a key role in stimulating the response of CD8^+^ T lymphocytes to viral antigens [17,30]. Intriguingly, CD169^+^ cells, like DCs, appear to serve as a crucial link between innate and adaptive immunity by facilitating antigen presentation, thereby contributing to the eradication of pathogens [31]. 

Understanding the pathogenesis of SARS-CoV-2 infection through the interplay of immne-inflammatory players meets the clinical need to identify biomarkers predictive of disease severity and prognosis for risk stratification and patient management. Given these observations, our study aimed to analyze, via multi-parametric flow cytometry, the frequency distribution and absolute number of peripheral blood CD169-expressing monocytes and those of conventional CD1c^+^ and CD141^+^ (namely cDC2 and cDC1) and plasmacytoid CD303^+^ DCs in a cohort of hospitalized patients affected by COVID-19 pneumonia. The prognostic impact of these immune-inflammatory cell players on in-hospital mortality was investigated. A gene profile analysis focused on 770 immune-inflammatory-related human genes and 20 SARS-CoV-2 genes was performed in a sub-group of cases to further address the host–SARS-CoV-2 interplay.

## 2. Materials and Methods

### 2.1. Study Population

Fifty-eight patients affected by COVID-19 pneumonia were prospectively enrolled between March and May 2021 upon hospital admission in our ward. Inclusion criteria were age ≥18 years, infection with SARS-CoV-2 confirmed via a positive reverse transcriptase polymerase chain reaction (RT-PCR) assay on a nasopharyngeal swab, and evidence of lung parenchyma involvement upon high-resolution chest computed tomography (CT). Patients with concomitant pulmonary embolism or ARDS were excluded. The study population’s demographics and clinical and laboratory data are shown in Table 1. Twenty-two sex- and age-matched SARS-CoV-2-negative healthy volunteers (M = 15; mean age ± SD = 63 ± 9.8 years), recruited among blood donors and patient family members, were enrolled as a control group for comparison. The study was conducted following the amended Declaration of Helsinki and approved by the local ethics committee (protocol number AOC/0000524/2020 on 26 June 2020). At enrolment, two peripheral blood samples (specified below) were collected via venipuncture from all study participants once written informed consent was obtained. In addition, data of interest for the study were anonymously collected into a dedicated database. Variables included socio-demographic characteristics, smoking habits, comorbidities, laboratory data, chest CT score of pneumonia in accordance with Chung et al. [32], any device used for oxygen supply, and pharmacological therapies. To date, oxygen supplementation has been administered to all patients (either via nasal cannula or vent mask). High-flow nasal cannula, and/or continuous positive pressure (CPAP) and/or non-invasive mechanical ventilation were required in 45 of them. All patients were treated with systemic steroids (either 0.5–0.75 mg/kg/day of methylprednisolone or 6 mg/day of dexamethasone) and low-molecular-weight heparin at a prophylactic dosage. Anti-viral therapies (namely remdesevir) were used in 15% of cases. 

### 2.2. Frequency Distribution and Absolute Numbers of Circulating CD169-Expressing Monocytes

Briefly, 50 μL of the EDTA-treated whole venous blood was incubated for 20 min in the dark at room temperature with a cocktail of monoclonal antibodies (mAbs), including anti-CD64 (Pacific Blue), anti-CD169 (PE), anti-DR (APC) and the anti-CD4 (APC-CY7). Matched isotype mAbs were used as negative controls. All reagents were purchased from Beckman Coulter (Brea, CA, USA). After erythrocyte lysis with ammonium chloride, 5 × 10^4^ events/sample were acquired using Navios Cytometer (Beckman Coulter, Brea, CA, USA) at the Clinical Biochemistry Unit of Monaldi Hospital. The analysis was performed using Kaluza C Software version 1.1. (Beckman Coulter, Brea, CA, USA). The morphological selection of monocytes was first based on forward and side light scatters (FSC-A and SSC-A, respectively). Gated cells were then plotted in accordance with CD64 expression/SSC. This way, the enumeration of CD169-expressing cells was restricted to CD64^+^ monocytes. The gating strategy for identifying CD169^+^ monocytes is shown in Supplementary Figure S1A. Data are expressed as percentages and absolute numbers. Absolute numbers were calculated as follows: percent of CD169^+^ monocytes × total number of white blood cells (WBCs) per mm^3^/100. The white blood cell (WBC) count was determined using a Sysmex XT-1800i hemocytometer (Sysmex Europe, Norderstedt, Germany).

### 2.3. Frequency Distribution and Absolute Numbers of Circulating Dendritic Cell Subsets

A flow cytometry DC enumeration kit (Blood Dendritic Cell Enumeration Kit, Milteny Biotech, Bergisch Gladbach, Germany) was used to identify DC subtypes in accordance with the manufacturer’s instructions. Reference median frequencies of blood DC subsets were 0.27% (range: 0.09–0.42), 0.02% (range: 0–0.04) and 0.19% (range: 0.09–0.37) for cDC2, cDC1 and pDC, respectively. Briefly, 300 μL of the EDTA-treated whole blood was incubated for 10 min with a cocktail of mAbs, including anti-CD1c (PE), anti-CD141(APC) and the anti-CD303 (FITC). Mouse IgG2a-PE, IgG1-FITC and IgG1-APC mAbs were used as isotype controls. In addition, samples were co-stained with anti-CD19-PE-Cy-5 and the anti-CD14-PE-Cy5 to exclude B cells and monocytes from the analysis. All specimens were treated with a dead cell discriminator. Following erythrocyte lysis, washing and fixation, 5 × 10^5^ events per sample were acquired using Navios Cytometer (Beckman Coulter, Brea, CA, USA) at the Clinical Biochemistry Unit of Monaldi Hospital and analyzed using Kaluza Software (Beckman Coulter, Brea, CA, USA). The gating strategy for identifying each DC subtype is shown in Supplementary Figure S1B. Results are reported as absolute numbers of cDC2, cDC1 or pDC among total WBCs. Data are expressed as percentages and absolute numbers. Absolute numbers were calculated as follows: percent of a given DC subset × total number of WBCs per mm^3^/100.

### 2.4. Measurement of Serum Levels of Interleukin-6

Serum levels of interleukin-6 (IL-6) were evaluated via a chemiluminescence immunoassay (Immulite 2000 System, Siemens Healthcare Diagnostics, Forchheim, Germany) at the Clinical Biochemistry Unit of Monaldi Hospital, immediately after blood sampling in all subjects, in accordance with the manufacturer’s instruction. The analytical sensitivity of the assay was 2 pg/mL, and the upper reference value in adults was 5.9 pg/mL.

### 2.5. Human Immune-Inflammatory and SARS-CoV-2 Gene Profile Analysis

For gene profile analysis, 2.5 mL of peripheral blood was collected into a PAXgene blood RNA tube. RNA was extracted using the RNA blood mini kit (Qiagen, Hilden, Germany), in accordance with the manufacturer’s instructions. Purified RNA was used for hybridization and subjected to gene profiling analysis on NanoString nCounter. An autoimmune profiling panel of 770 human genes involved in immune-inflammatory processes were tested as target. Coronavirus Panel Plus containing 20 probes for the SARS-CoV-2 genes (ACE2_Hs; HCoV-229E_N; HCoV-229E_S; HCoV-HKU1_N; HCoV-HKU1_S; HCoV-NL63_N; HCoV-NL63_S; HCoV-OC43_N; HCoV-OC43_S; SARS-CoV_N; SARS-CoV_S; SARS-CoV-2_E; SARS-CoV-2_M; SARS-CoV-2_N; SARS-CoV-2_orf1ab; SARS-CoV-2_orf1ab_REV; SARS-CoV-2_ORF3a; SARS-CoV-2_ORF7a; SARS-CoV-2_ORF8; SARS-CoV-2_S) was analyzed as well. Gene expression data were normalized using nSolver Version 4.0 Software with reference to internal ERCC (External RNA Controls Consortium) technical controls and 30 housekeeping genes. Statistical analysis was performed via the Benjamini–Hochberg procedure. We conducted heatmap analyses to correlate the most important covariates.

### 2.6. Statistical Analysis

Quantitative variables were characterized using the mean ± standard deviation (SD) or median with quartile ranges, where appropriate. Categorical factors were described as absolute numbers and percentages. Comparisons between study groups were analyzed with the Mann–Whitney U test. Correlations among quantitative variables were based on the non-parametric Spearman rank correlation coefficient. Survival was calculated using Kaplan–Meier curves and compared via the log-rank test. In-hospital mortality was calculated from the date of hospital admission to the date of death. All tests were two-tailed, and statistical significance was set at *p* < 0.05. Statistical analyses were conducted using Qlucore software 3.9, GraphPad Prism 8 (GraphPad Software Inc., San Diego, CA, USA) or MedCalc (MedCalc Software 22.023, Ostend, Belgium).

## 3. Results

### 3.1. Differential Distribution of Circulating CD169^+^ Monocytes and Dendritic Cell Subsets in COVID-19 Pneumonia

**Figure 1:**
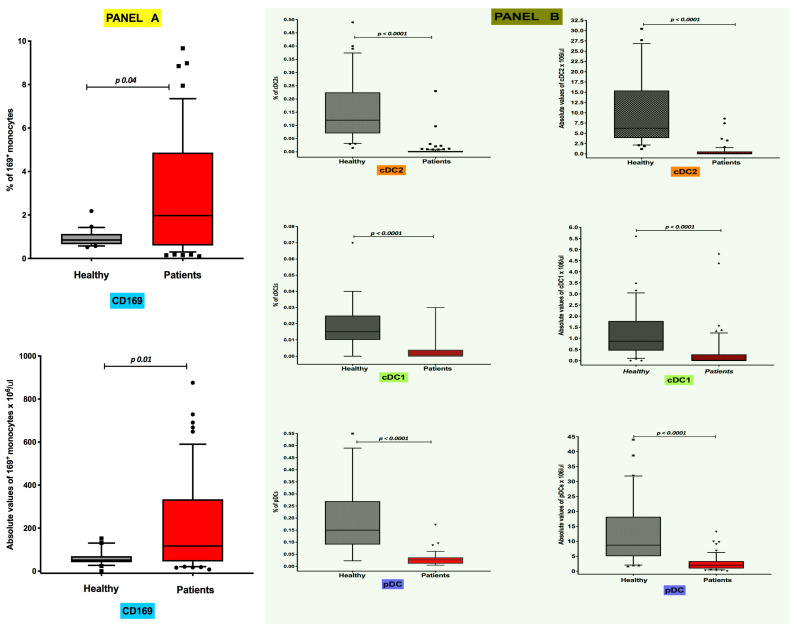
Highs levels of CD169+ monocytes and low values of circulating DC subsets are present in the peripheral blood of COVID-19 patients. Boxplot showing the percentage distribution and absolute counts of monocytes CD169^+^ (**A**) and circulating cDC2, cDC1 and pDC (**B**) subsets among total leukocytes in healthy subjects and COVID-19 patients.

Flow cytometry estimates of median frequencies and absolute levels of peripheral blood CD169-expressing monocytes were significantly higher in COVID-19 patients than in healthy controls. In particular, the median frequency values [25th; 75th percentile] were 0.85% [0.65; 1.13] in healthy individuals and 1.99% [0.58; 4.92] in COVID-19 patients (*p* 0.04) (Figure 1, Panel A), while the median absolute levels were 51.4 cells/μL [41.3; 69.7] and 116.9 cells/μL [43.4; 346.1], respectively (*p* 0.01) (Figure 1, Panel A). Conversely, as reported in Figure 1 (Panel B), both frequencies and absolute median counts of all DC subsets were severely depleted in COVID-19 patients. Specifically, the median frequencies in healthy controls were 0.120% [0.070; 0.225] for CD1c^+^ cDC2s, 0.0150% [0.010; 0.025] for CD141^+^ cDC1s and 0.150% [0.090; 0.270] for CD303^+^ pDCs (Figure 1, Panel B), while those in in COVID-19 patients were 0.000% [0.000; 0.003] for CD1c^+^ cDC2s, 0.000% [0.000; 0.004] for CD141^+^ cDC1s and 0.0270% [0.011; 0.037] for CD303^+^ pDCs (Figure 1, Panel B). The median absolute numbers of DC subsets in healthy individuals were 6.226 cells/μL [3.85; 15.43] for CD1c^+^ cDC2s, 0.870 cells/μL [0.87; 1.78] for CD141^+^ cDC1s and 8.721 cells/μL [5.02; 18.21] for CD303^+^ pDCs (Figure 1, Panel B), while DC absolute counts in COVID-19 patients were 0.00 cells/μL [0.00; 0.58] for CD1c^+^ cDC2s, 0.00 cells/μL [0.00; 0.28] for CD141^+^ cDC1s and 2.01 cells/μL [0.91; 3.38] for CD303^+^ pDCs (Figure 1, Panel B). For all the DC subsets, differences compared with the control group were equally statistically significant in all comparisons (*p* < 0.0001). 

### 3.2. Inverse Correlation of Absolute Levels of CD169-Positive Monocytes with CD141^+^ cDC1 Numbers in COVID-19 Pneumonia Patients

Perturbations at the baseline of cDC1s and pDCs were inversely correlated with CD169-positive monocytes (r = −0.29 and *p* = 0.0309, and r = −0.31 and *p* = 0.0347, respectively). 

Moreover, pDC absolute values were significantly correlated with lymphocytes (r = 0.46; *p* = 0.0001) and monocytes (r = 0.44; *p* = 0.0003). No further correlations were found between the peripheral cell subtypes analyzed and the patients’ clinical and laboratory data. 

### 3.3. Short-Term Distribution of Circulating CD169^+^ Monocyte DC Subsets Is Not Modified by Systemic Steroids

To address the impact of systemic steroids on the peripheral distribution of CD169-expressing monocytes and DC subsets, a sub-cohort of 20 COVID-19 patients treated with systemic steroids were tested 7 and 14 days from hospital admission. Absolute numbers of both CD169^+^ monocytes and DC subsets remained unchanged at the time points analyzed. 

### 3.4. Circulating CD169^+^ Monocytes Are Associated with In-Hospital Mortality from COVID-19 Pneumonia

**Figure 2:**
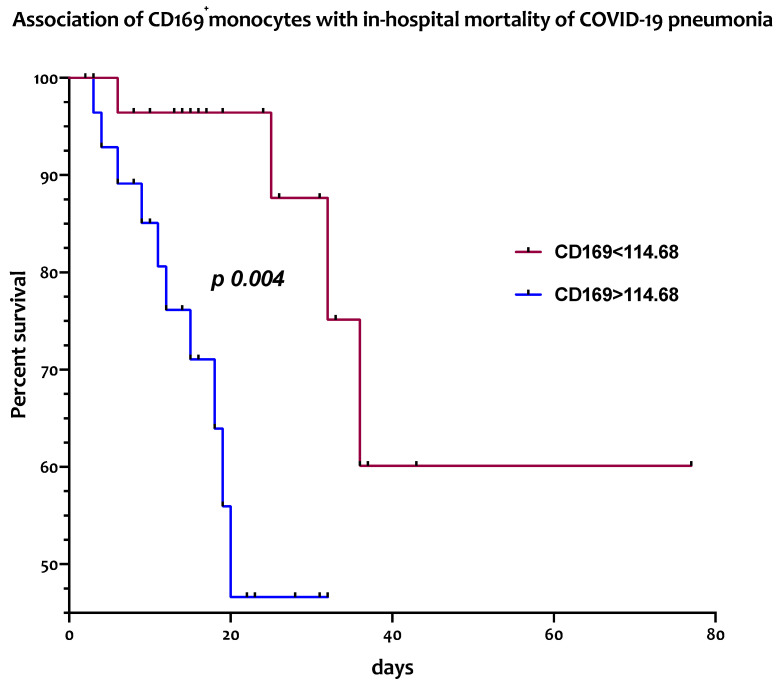
High numbers of peripheral CD169^+^ monocytes are associated with increased in-hospital mortality in COVID-19 patients Kaplan–Meier curves representative of the in-hospital mortality of COVID-19 patients according to the high (>cut-off) or low (<cut-off) baseline levels (T0) of circulating CD169^+^ monocytes.

The association between the frequencies and absolute numbers of circulating CD169-positive monocytes and of DC subsets with in-hospital mortality was assessed with a log rank test. As reported in Figure 2, patients affected by COVID-19 pneumonia with absolute counts of peripheral CD169^+^ monocytes above the median value of 114.68/μL hadsignificantly higher in-hospital mortality (median survival of 20 days) [HR 4.33; 95% CI: 1.46–12.88] than those with lower values (median survival >75 days) [HR 0.23; 95% CI: 0.07–0.68; *p* = 0.004]. 

Conversely, no significant differences in in-hospital mortality were observed between patients when looking at frequencies of CD169^+^ monocytes or percentages and absolute levels of any circulating DC subset.

### 3.5. Serum Levels of Pro-Inflammatory Cytokine IL-6 Are Increased in COVID-19 Patients and Inversely Correlated with the Absolute Numbers of cDC1s and pDCs

**Figure 3:**
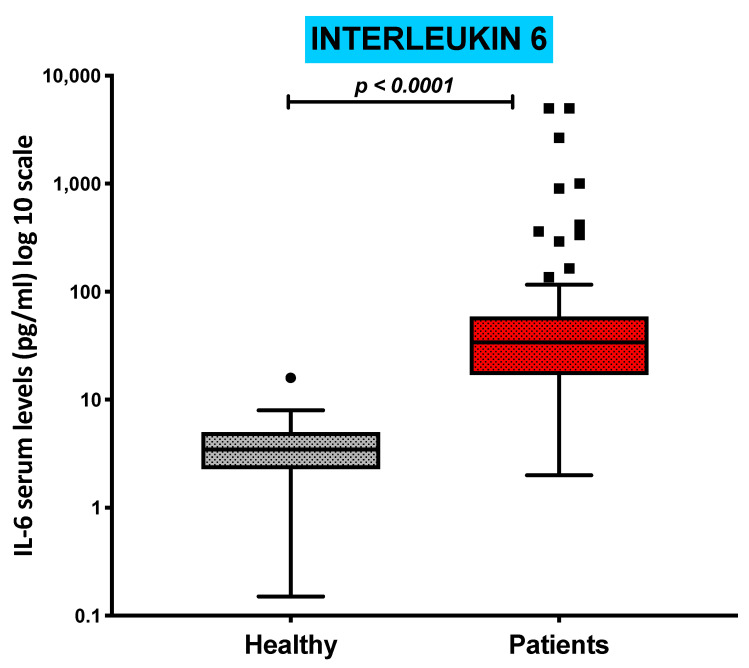
Serum concentrations of interleukin-6 are increased in COVID-19 patients. Boxplot showing the distribution of Interleukin-6 analyzed in healthy subjects and COVID-19 patients.

Serum levels of the pro-inflammatory cytokine IL-6 were measured in all patients to assess the COVID-19-related peripheral immune-inflammatory milieu. IL-6 concentrations were significantly increased in COVID-19 patients compared with those of the control group (*p* < 0.0001), as shown in Figure 3, ranging from a minimum of 2.00 pg/mL to a maximum of 5000 pg/mL.

Correlation analysis between IL-6 serum levels and the distribution of all DC subtypes further showed that in COVID-19 patients, IL-6 was negatively relatedto the circulating absolute levels of the CD1c^+^ cDC2 subpopulation (r = −0.29, *p* = 0.034) and CD303^+^ pDC subsets (r = −0.29, *p* = 0.036). No significant correlation was observed with CD141^+^ cDC1 or the expression of CD169-expressing monocytes.

### 3.6. Interplay of Host Immune-Inflammatory Genes and SARS-CoV-2 Gene Signature in COVID-19 Pneumonia

**Figure 4:**
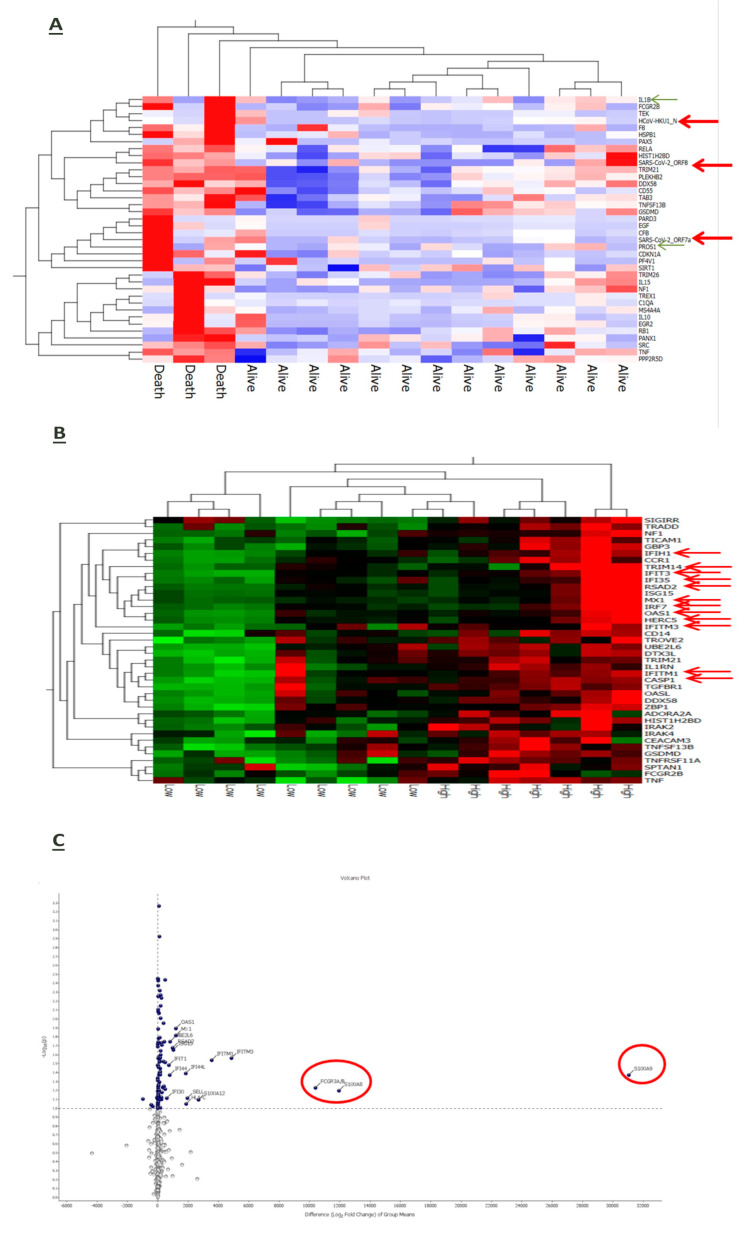
Gene expression analysis of baseline samples. The red arrows indicate the viral genes. The green arrows indicate pro-inflammatory and anticoagulant genes (**A**). In patients stratified in accordance with IL-6 levels, the red arrows indicate genes involved in the innate antiviral response induced by interferon and inflammatory cytokines (**B**). In terms of fold changes, there are also several genes related to the inflammation and interferon gamma pathway (**C**).

An RNA-based gene profiling analysis of a panel of 770 human genes involved in immune-inflammatory processes was available for a sub-cohort of 16 patients at enrollment upon hospital admission. Three of them died during the observation period due to a sudden deterioration of their clinical conditions. Interestingly, the expression of some viral genes (HCoV-HKU1_N, SARS-CoV2_ORF8 and SARS-CoV2_ORF7a) was upregulated in patients with worse outcomes along with inflammatory mediators, such as interleukin (IL)-1 beta, tumor necrosis-α (TNF-α) and the anticoagulant protein (PROS1) (Figure 4A). Also, stratification of the whole patient sub-group in accordnace with the median reference value of IL-6 serum levels (31.46 pg/mL) allowed us to identify seven cases in whom higher IL-6 expression was associated with the upregulation of genes involved in the anti-viral response induced by interferon, such as *IFIH1*, *IFIT3*, *IFI35*, *IRF7*, *IFITM3* and *IFITM1*, and of the inflammatory cytokines S100A9 and S100A8 (Figure 4B) and (Figure 4C).

**Figure 5:**
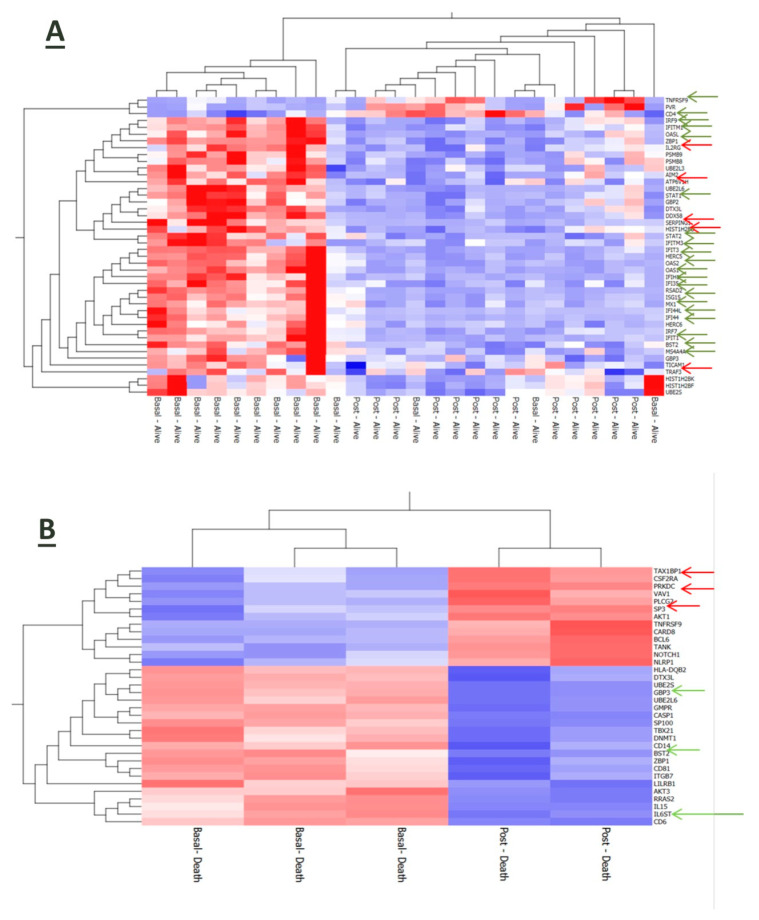
Gene profile analysis of baseline versus on-treatment samples (**A**). The green arrows indicate genes involved in the inflammatory and interferon pathway. The red arrows indicate immunity-related genes. (**B**) Gene profile analysis of baseline versus on-treatment samples in the group of dead patients. The green arrows indicate inflammation-related genes. The red arrows indicate genes related to antiviral activity (**B**).

Even after 7 days from hospitalization, immune-inflammatory gene expression was modulated in accordance with the disease outcome of COVID-19 patients. In particular, surviving patients’ upregulated genes were related to inflammatory and anti-viral-related pathways along with the T cell membrane molecule CD4 (Figure 5A). Conversely, patients who died had a reduced expression of genes involved in anti-viral defense (Figure 5B). 

## 4. Discussion

The pathogenesis of COVID-19 remains a puzzle that has not been fully clarified and that involves the contribution of multiple host-related immune factors. 

The aim of our study was to assess the interplay between circulating CD169^+^ monocytes and DCs, which are part of the monocyte–phagocyte system and key players in the modulation of the immune-inflammatory process against viral infections [12,25], in a cohort of patients hospitalized for COVID-19 pneumonia. In agreement with previous data, our study confirms the involvement of the MPS in COVID-19 pneumonia through the finding of a lack of balance between CD169^+^ monocytes (increased) and dendritic cell subsets (severely depleted) at the peripheral level. This observation was significantly associated with a worse disease outcome (in-hospital mortality). Interestingly, in line with cellular findings, gene profiling analysis revealed a panel of inflammation-related host products, including interleukin-6, which notably exerts a suppressive effect on DC maturation, along with viral genes being over-expressed in patients with bad prognosis. 

CD169-expressing monocytes are present in high numbers in the spleen and lymph nodes, where they are involved in autoimmunity and defense against viral infections. This happens upon stimulation with interferons (IFNs), which typically increase their constitutive low expression of CD169 [33,34,35]. Therefore, the overexpression of CD169 may be considered a promising host marker of viral infections. Within a pandemic context, this possibility has ignited interest in CD169-expressing monocytes as a rapid marker for the triage of patients with suspected COVID-19. Also, there is evidence that CD169^+^ monocytes display a distinct gene expression profile and are functionally different in COVID-19 patients [36]. We found that both the proportion and the absolute number of CD169^+^ monocytes were increased in our patient cohort. Interestingly, in-hospital mortality was increased in cases with circulating CD169^+^ monocytes above the median reference value. Similarly to our setting, previous studies have shown that blood CD169^+^ monocytes were significantly increased in COVID-19 cases, correlating with disease severity [12,37]. In this direction, Ortillon et al. have shown that CD169-expressing monocytes were more activated in COVID-19 patients with bad prognosis requiring mechanical ventilation [38]. Despite this, the relationship of CD169^+^ monocytes with patient outcomes still remains not univocal in COVID-19. Indeed, lower numbers of CD169^+^ monocytes have been equally related with poorer prognosis in COVID-19 patients [27,39], while other observations have reported no association [22]. 

Herein, we also show that the increase in CD169^+^ monocytes was associated with a severe drop in all circulating DC subsets, either analyzed as frequencies or absolute numbers. This combined observation is the first to be reported to emphasize the close complementarity between these two different cell types. Previous studies have reported that the numbers of cDCs and pDCs were lower in COVID-19 subjects than in healthy individuals [40,41,42,43], and that DC impairment was restored in convalescent phases [40]. Among the different DC subsets, pDCs display high antiviral activities due to their ability to produce IFN-I and are thought to be involved in immune tolerance [44]. By virtue of these properties, pDCs, similarly to CD169-expressing monocytes, have been suggested as a biomarker of COVID-19, strongly related with disease severity [39]. The so-called cytokine storm that characterized the most severe forms of COVID-19 was an exuberant inflammatory response associated with high levels of serum IL-6 [23,45]. Cumulative evidence suggests that IL-6 inhibits the differentiation of DCs by affecting the transition from the resting/immature phenotype to the activated/mature one through the IL-6–JAK2–STAT3 axis, which is emerging as a major player in inflammation [46,47]. In our series, we found that serum concentrations of the pro-inflammatory cytokine IL-6 were significantly higher in COVID-19 patients than in healthy controls. Also, IL-6 levels negatively correlated with the absolute number of both pDCs and cDCs, suggesting the close relationship of inflammation with the impairment of anti-viral responses and the initiation of adaptive immunity. According to these data, the ancillary human gene profiling analysis performed in a sub-cohort of our patient population also revealed that inflammation-related gene products, such as IL-1 beta, TNF-α and the anticoagulant protein (PROS1), were over-expressed in COVID-19 patients along with some viral genes (HCoV-HKU1_N, SARS-CoV2_ORF8 and SARS-CoV2_ORF7a). In addition, as far as our study could be considered a qualitative assessment, the increased expression of IL-6 was found to be associated with the upregulation of genes involved in the innate antiviral response, such as IFIH1, IFIT3, IFI35, IRF7, IFITM3 and IFITM1, and of the inflammatory cytokines S100A9 and S100A8. Of note is that patients who died had a reduced expression of genes involved in anti-viral defense.

Our study has some limitations. First, it was conducted on a small cohort of patients from a single center. This means that results cannot be generalized and no consideration can be given for the impact of treatments. Unfortunately, the restrictions imposed by the pandemic have strongly penalized experimental studies on potentially contagious samples. Also, the short period of the study was representative of a clinical picture that has changed over time as a result of the virus’ gene variability. A further limitation is represented by the unavailability of virus genotyping at the time of the study. However, our data are noteworthy due to the fact that the enumeration and phenotypic analysis of CD169-expressing cells and DC subtypes suffer from low values of the latter at the peripheral level, even in healthy individuals. In addition, the use of high-cost molecular analytical procedures was a further constraint on the extension of the study to a larger case study in an extremely difficult context. In this issue, our effort represents a precious piece of the intriguing puzzle of the immuno-pathology of SARS-CoV-2 infection. 

The dramatic scenario of the SARS-CoV-2 pandemic has immediately imposed the need to understand the pathogenetic mechanisms, and from these, the need to take advantage of biomarkers of clinical use. Since the early stages of the pandemic, parameters of prompt availability such as ratios between neutrophils or D-dimer and lymphocytes were representative of an exuberant and uncontrolled immune-inflammatory reaction. In an attempt to explore the mechanisms involved [48,49], our findings suggest that the interplay between the different components of the MPS is dysregulated in COVID-19 patients presenting with acute pneumonia. An excessive inflammatory response together with the recruitment of cells of natural immunity (that is, an increased availability of CD169-expressing monocytes) is at the same time a cause of disease progression but also an attempt to compensate for the impairment of the anti-viral and adaptive responses (that is, the depletion of DC subsets). This may explain, at least in part, the imbalance between innate and adaptive immunity and its impact on disease outcomes, and offer the possibility of including new biomarakers for risk stratification and disease prognosis in clinical practice. Although the clinical manifestations of COVID-19 have significantly changed, even in the face of a tight vaccination campaign, a deepening of the pathogenesis mechanisms underlying a condition that has devastated the planet remains a priority of clinical and experimental research.

## Figures and Tables

**Table 1:** Parameter.

Parameter	
Participants, *n*	58
Age (yrs)	68 ± 12.8
Sex (M/F)	40/18
Smoking habit (either active or former)	33 (58)
Body mass index (kg/m^2^)	27.3 [25–30.8]
Type II diabetes	34 (58)
Systemic arterial hypertension	33 (58)
Chest CT Chung score	13 [8–15]
paO_2_/FiO_2_	122.5 [96–170]
neutrophils/lymphocytes	13 [5.6–24.1]
C reactive protein (mg/dL)	4.9 [2.1–9.4]
Interleukin-6 (pg/mL)	34 [17–59]
KL-6 (U/mL)	657 [349–1354]
D-Dimer (μg/mL)	422 [250–937]
Lenght of hospitalization (days)	16 [10.75–24.25]

Data are expressed as mean ± standard deviation, median [IQR25–IQR75], and number (%).

## Data Availability

The datasets used and analyzed during the current study are available from the corresponding author upon reasonable request.

## Supplementary Materials

The following supporting information can be downloaded at https://www.mdpi.com/article/10.3390/jcm13092481/s1: Figure S1: Gating strategy for CD169^+^ monocyte identification. (A) Initial monocyte selection utilized FSC-A and SSC-A parameters, followed by gating on CD64 expression/SSC. Subsequent analysis focused exclusively on CD169^+^ cells within the CD64^+^ monocyte population, as demonstrated in the gating strategy; (B) Gating strategy for dendritic cell subset identification. Low SSC and non-CD19/CD14-expressing cells were selected in order to exclude monocytes, B cells and granulocytes. Selected cells were plotted in accordance with CD141 and CD303 expression (C,D) to identify cDC1s and pDCs, respectively. Further plotting in accordnace with CD1c and CD303 expression allowed the identification of cDC2s (E,F).
