# Supplementary figures and images for: The Dysregulation of the Monocyte–Dendritic Cell Interplay Is Associated with In-Hospital Mortality in COVID-19 Pneumonia
Source: J Clin Med. 2024 Apr 24;13(9):2481. doi: 10.3390/jcm13092481 (PMC11084469; doi:10.3390/jcm13092481)

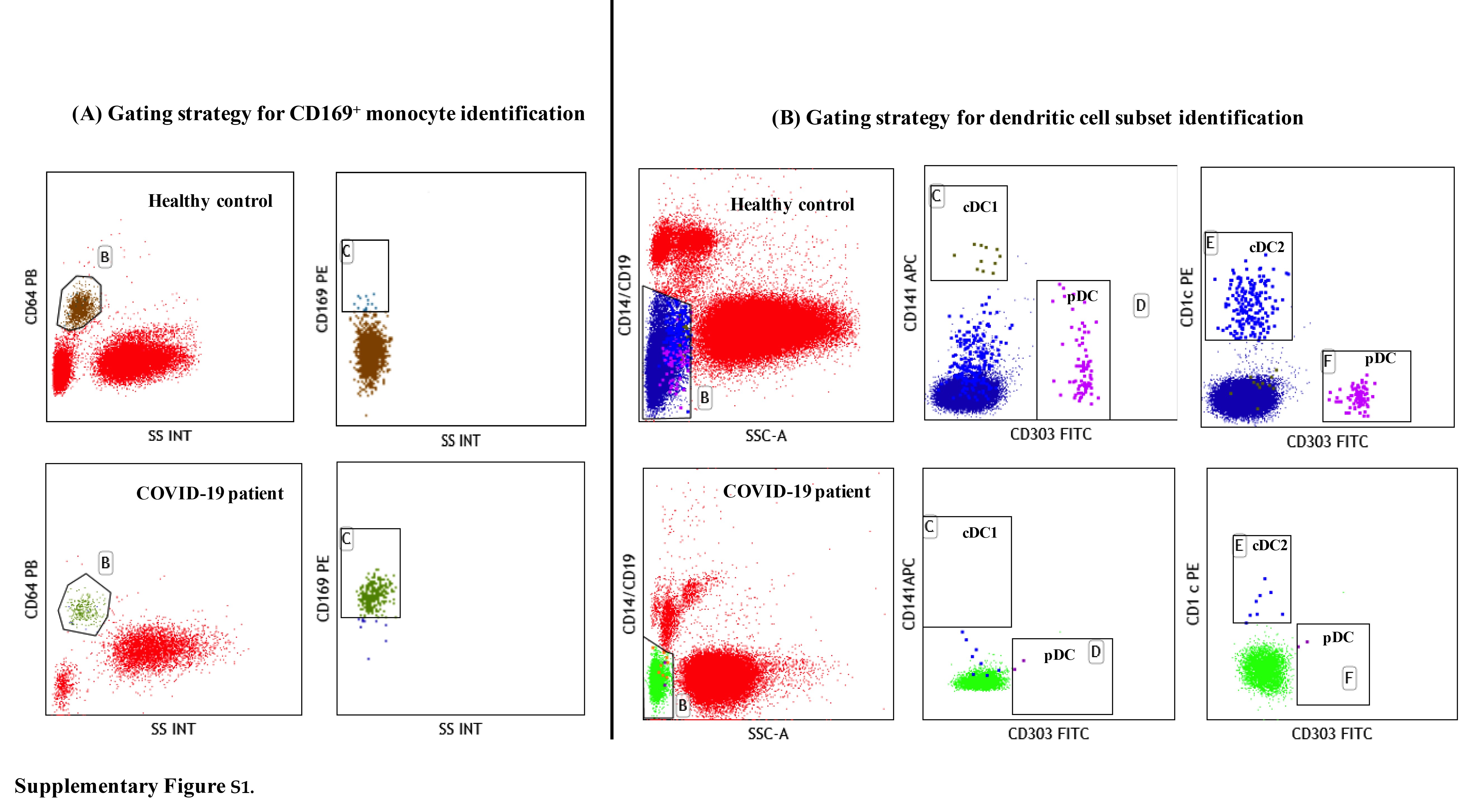

Supplement: Supplementary file 1 [file jcm-13-02481-s001.zip › jcm-2942029-supplementary.jpg]
